# Indomethacin injury to the rat small intestine is dependent upon biliary secretion and is associated with overgrowth of enterococci

**DOI:** 10.14814/phy2.12725

**Published:** 2016-03-31

**Authors:** Sara A. Mayo, Ye K. Song, Melissa R. Cruz, Tri M. Phan, Kavindra V. Singh, Danielle A. Garsin, Barbara E. Murray, Elizabeth J. Dial, Lenard M. Lichtenberger

**Affiliations:** ^1^ Departments of Integrative Biology & Pharmacology, Microbiology and Molecular Genetics, and Internal Medicine‐Infectious DiseasesThe University of Texas Health Science Center at HoustonHoustonTexas

**Keywords:** Bile acid, *Enterococcus faecalis*, non‐steroidal anti‐inflammatory drugs

## Abstract

NSAID use is limited due to the drugs’ toxicity to the gastrointestinal mucosa, an action incompletely understood. Lower gut injury induced by NSAIDs is dependent on bile secretion and is reported to increase the growth of a number of bacterial species, including an enterococcal species, *Enterococcus faecalis*. This study examined the relationships between indomethacin (INDO)‐induced intestinal injury/bleeding, small bowel overgrowth (SBO) and dissemination of enterococci, and the contribution of bile secretion to these pathological responses. Rats received either a sham operation (SO) or bile duct ligation (BDL) prior to administration of two daily subcutaneous doses of saline or INDO, and 24 h later, biopsies of ileum and liver were collected for plating on selective bacterial media. Fecal hemoglobin (Hb) and blood hematocrit (Hct) were measured to assess intestinal bleeding. Of the four treatment groups, only SO/INDO rats experienced a significant 10‐ to 30‐fold increase in fecal Hb and reduction in Hct, indicating that BDL attenuated INDO‐induced intestinal injury/bleeding. Ileal enterococcal colony‐forming units were significantly increased (500‐ to 1000‐fold) in SO/INDO rats. Of all groups, only the SO/INDO rats demonstrated gut injury, and this was associated with enterococcal overgrowth of the gut and dissemination to the liver. We also demonstrated that INDO‐induced intestinal injury and *E. faecalis* overgrowth was independent of the route of administration of the drug, as similar findings were observed in rats orally dosed with the NSAID. Bile secretion plays an important role in INDO‐induced gut injury and appears to support enterococcal overgrowth of the intestine. NSAID‐induced enterococcal SBO may be involved either as a compensatory response to gut injury or with the pathogenic process itself and the subsequent development of sepsis.

## Introduction

Nonsteroidal anti‐inflammatory drugs (NSAIDs) are widely used to treat pain and inflammation, but are limited in their usefulness due to their ability to cause gastrointestinal bleeding (Blackler et al. 2014). Nonaspirin NSAIDs, such as indomethacin (INDO), most of which are secreted into the bile, are known to induce severe injury to the lower gut, specifically the distal small intestine which may result in perforation and obstruction in both rodents and humans (Wax et al. 1970; Beck et al. 1990; Jacob et al. 2007; Dial et al. 2008; Wallace 2013). NSAID‐induced ulceration of the GI mucosa allows for overgrowth of normal gut bacteria in the small intestine of both man and animals (Reuter et al. 1997; Kim et al. 2005; Muraki et al. 2014), and increases the probability of bacterial dissemination throughout the body. The presence of *Enterococcus faecalis*, a normal inhabitant of the intestine but also a common cause of nosocomial infections, has been shown to increase dramatically and somewhat specifically in the rat gut with use of the NSAID, INDO (Dalby et al. 2006). Although an association between gut injury and bacterial overgrowth has been shown previously, their relationship is unclear. Previous research using germfree rats has shown that the presence of intestinal bacteria are required for NSAID‐induced injury (Robert and Asano 1977). In addition, experimentally restricting the flow of bile while using an NSAID can lessen ulceration and injury (Brodie et al. 1970; Somasundaram et al. 1997; Jacob et al. 2007; Lichtenberger et al. 2011). The fact that *E. faecalis* and other enterococci grow in medium supplemented with bile acids could offer an explanation for the association of NSAID‐induced injury with intestinal bacteria and the presence of bile in the GI lumen. It was the aim of this study to investigate the importance of bile acid by the use of bile duct–ligated rats, on INDO‐induced GI injury and overgrowth of potentially pathogenic species of enterococci.

## Materials and methods

### Chemicals

INDO purchased from Sigma Aldrich (St Louis, MO) was dissolved in phosphate‐buffered saline by adjustment of the pH to alkalinity and then lowering the pH below 8 prior to the experiment.

### Animal studies

All studies were approved by The University of Texas Health Science Center's Animal Welfare Committee, and all procedures done conformed to their regulations. Male Sprague–Dawley rats were purchased from Harlan Labs (approximately 200 g from Houston, TX).

For the bile‐duct ligation study, animals were fasted before surgery, anesthetized under isoflurane, and a right lateral incision was made; the duodenum and bile duct were visualized. For the bile duct ligation (BDL) groups, the common bile duct was blocked with a suture tie and for the sham‐operated (SO) groups, a loose suture tie was placed around the bile duct that did not constrict bile flow or vascular circulation. The abdominal incision was closed and the rats were allowed to recover with chow and water ad lib. At 24 h after surgery, either saline or 7.5 mg/kg of INDO was subcutaneously injected to comprise the final groups: SO/saline, SO/INDO, BDL/saline and BDL/INDO (*N* = 6–8/group). This injection was repeated again 24 h later, meaning that each rat received two doses of either saline or indomethacin. Then 24 h after the last dose and immediately prior to euthanasia, cardiac blood was collected under anesthesia into heparinized capillary tubes for standard determination of hematocrit (Hct) as one measure of intestinal bleeding. To assess bacterial gut overgrowth and dissemination, the liver was removed first followed by the small intestine to prevent seeding of bacteria from the gut to other organs. A sample of feces was removed from the large intestine for analysis of bacterial content and hemoglobin (Hb) concentration as another measure of intestinal bleeding. The small intestine was inspected macroscopically for mucosal injury (perforations, adhesions, lesions) and was flushed with ice‐cold saline, which was analyzed for total bile acid to confirm completeness of bile duct ligation and for bacterial samples for further identification by hybridization. A portion of ileum was set aside for pathology and the remainder of ileum, liver, and feces were weighed and then homogenized in 1 mL chilled 0.9% saline. Ten‐fold serial dilutions were made of homogenates and 25 *μ*L of each dilution was plated onto brain heart infusion agar (BHI) which is a culture medium for all bacteria, MacConkey's agar (MAC) which is a culture medium for gram‐negative bacteria, and bile esculin agar (BEA) or Enterococcosel^™^ agar (EA) both selective growth media for *Enterococcus* species due to the presence of 4% bile (oxgall) in the agar. Bacterial colony‐forming units (CFUs) were counted after 24‐h incubation at 37°C, and used to calculate the total number of CFU per g of tissue as a measure of direct colonization in the gut and dissemination to other organs.

The BDL studies were performed with indomethacin injected parenterally (i.e., subcutaneous) so that the presence of indomethacin in the gut would be restricted to that which was secreted through bile fluid. For comparison, another group of rats were treated with indomethacin administered orally with drug exposed directly to the gut, as well as through recirculation in the bile. Tissue collection and processing were as described above.

### Hybridization method

Animals (SO/INDO, BDL/INDO, SO/saline, and BDL/saline) were euthanized, lumen contents were flushed with 0.9% saline and samples were saved on ice for further processing and bacteria recovery. Ten‐fold serial dilutions of lumen wash samples were made in chilled saline and 25 *μ*L of each dilution was plated onto EA to select for enterococci growth. After ~48 h, up to 20 CFU/rat were picked into microtiter plate wells containing Brucella broth + 15% glycerol and/or using saline suspension made from previously EA grown colonies and were replica plated on to Hybond^™^ ‐N+ membranes (GE Healthcare) which had been placed on EA agar, then grown overnight and processed for hybridization as previously described (Singh et al. 1998), using intragenic DNA probes which were specific for *E. faecium* (*ddl* probe*)* (Dutka‐Malen et al. 1995), *E. faecalis* (*ace* probe) (Nallapareddy et al. 2000), *E. durans* (***mur*** **‐2**_**ed**_ probe) (Arias et al. 2006), and *E. hirae* (***mur‐2*** probe) (Arias et al. 2006).

### Biochemical assays

Bile acid was quantified using a Total Bile Acids Assay kit (DZ092A from Diazyme Laboratories, Poway, CA) according to the manufacturer's instructions. The assay for hemoglobin in feces has been described previously (Crosby and Furth 1956; Barrios and Lichtenberger 2000).

### Statistics

Values for hematocrit, hemoglobin, and bile acid are expressed as the mean ± standard error of the mean for *N* = 6–8 rats. Statistical differences for these assays were evaluated by ANOVA followed by Tukey's HSD method and assuming a significance level of 5%. Values for bacterial counts are expressed as log_10_ CFUs per gram of tissue. Due to a large positive skewing of recovery values noted by us and others (Hagberg et al. 1984), the data were log‐transformed and the unpaired *t* test used for statistical calculations. The geometric mean was used in graphical presentations.

## Results

### Effect of parenteral indomethacin on intestinal bleeding

**Figure 1:**
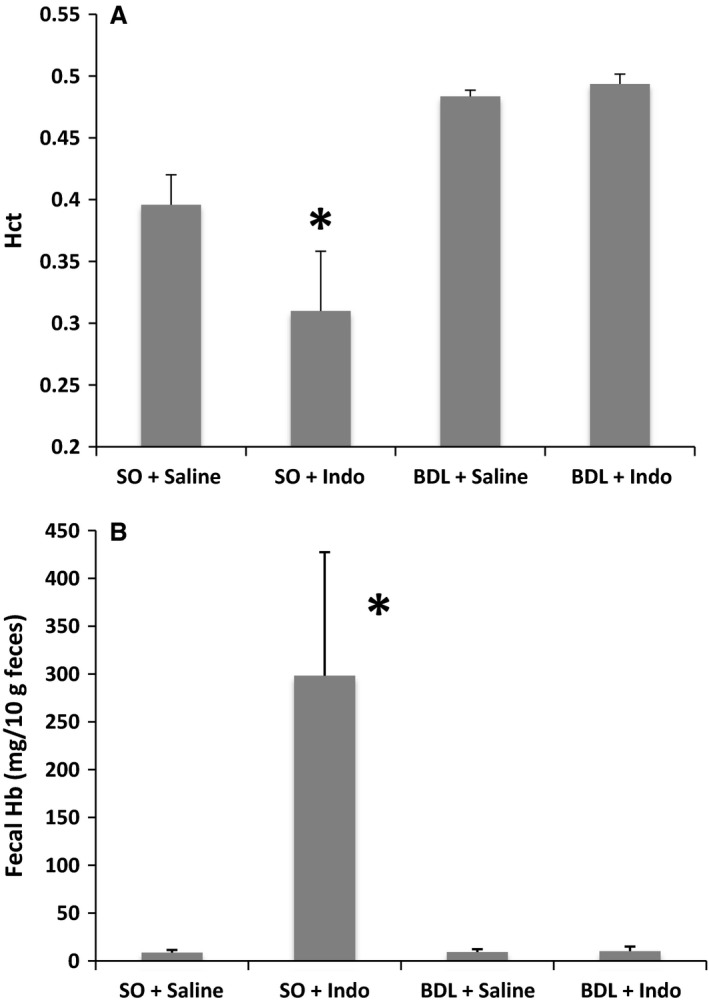
Assessment of GI bleeding due to experimental treatments was determined at the time of euthanasia by measurement of: (A) Hematocrit, and (B) Hemoglobin in feces. Both measures showed a difference only in the SO + INDO group. Values are expressed as the mean ± standard error of the mean. *P < 0.05 versus SO + saline and BDL groups.

The effectiveness of BDL surgery was found to be nearly complete, as all rats undergoing BDL had intestinal bile acid concentrations that were a fraction of SO animals (SO/saline = 6657 ± 1619 μmol/L; SO/INDO = 6090 ± 1599 μmol/L; BDL/saline = 502 ± 190 μmol/L; BDL/INDO = 571 ± 187 μmol/L). Hematocrit (Hct) was measured for each of the four groups to compare the degree of blood loss from intestinal injury. Results, as shown in Figure 1A, demonstrate that BDL attenuated gut injury in the presence of INDO. That is, rats treated with INDO that did not receive BDL (SO/INDO) had a reduced Hct when compared with the other groups (*P* < 0.05). Additionally, the hemoglobin (Hb) concentration measured in fecal samples illustrated consistent results (Fig. 1B). Overall, fecal Hb was markedly and significantly elevated in the SO/INDO group, whereas it was not increased in any of the other groups. In fact, the BDL/INDO group had comparable levels of Hb to the SO/saline and BDL/saline groups, showing that BDL attenuated gut injury in the presence of the NSAID.

### Effect of indomethacin on small intestine mucosal architecture in presence of bile duct ligation

**Figure 2:**
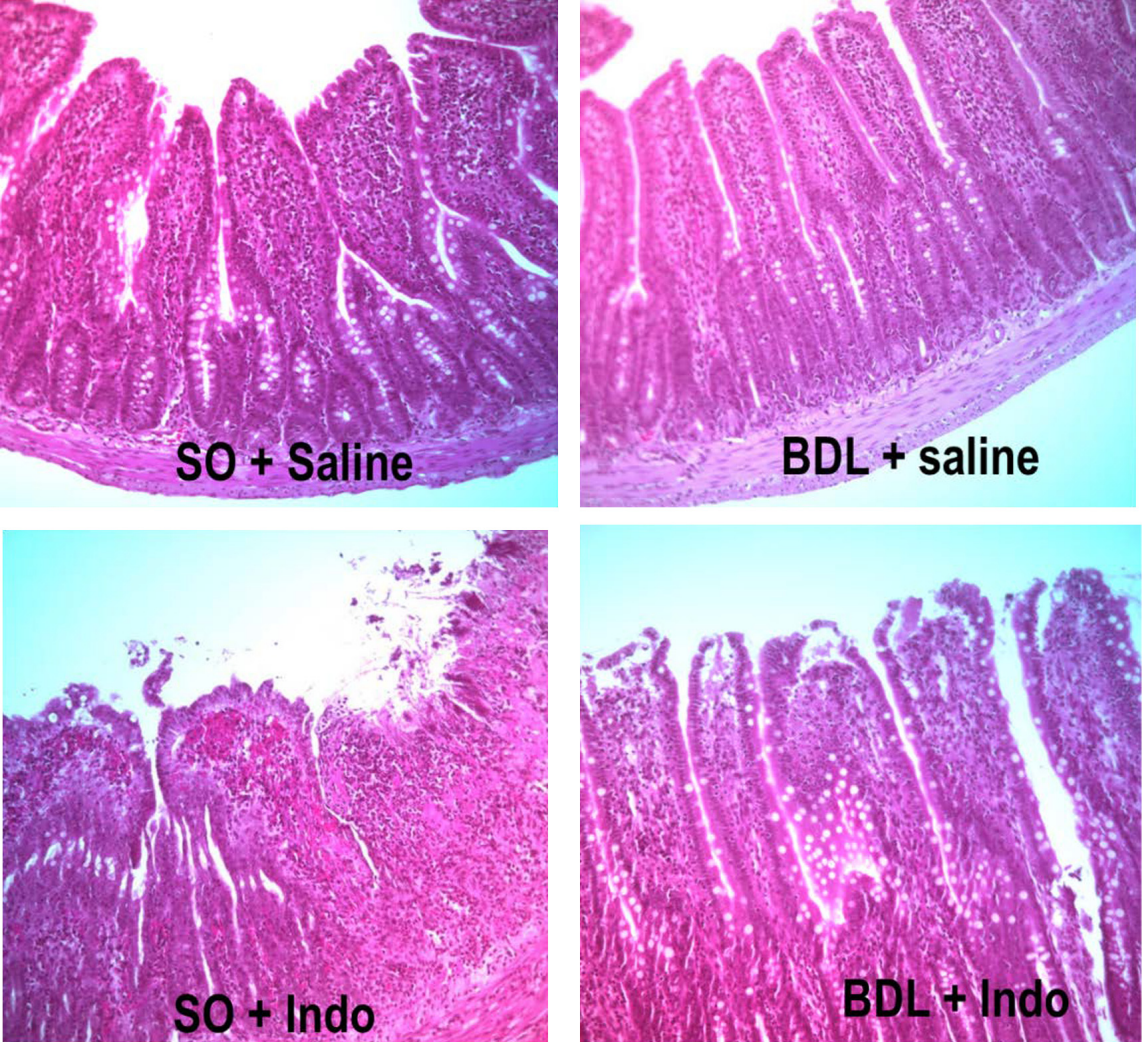
Histology of the ileum was performed on tissue samples collected at the time of euthanasia. Representative samples from each of the four treatment groups are shown at the same 40× magnification. Staining was with H&E.

During removal of the small intestine from the rats it was possible to see (macroscopically) punctate lesions indicating bleeding only in the lumen of the sham‐operated rats treated with indomethacin (SO/INDO). When viewed microscopically with H&E stained sections (Fig. 2), it was seen that indomethacin caused damage to the ileal villi in the SO/INDO group. Some surface damage was also seen in the gut of the BDL/INDO group with some evidence of intra‐villus edema, however, the overall architecture of the villi was preserved and there was no cell sloughing into the lumen which was not the case for the SO/INDO group.

### Intestinal overgrowth with indomethacin

**Figure 3:**
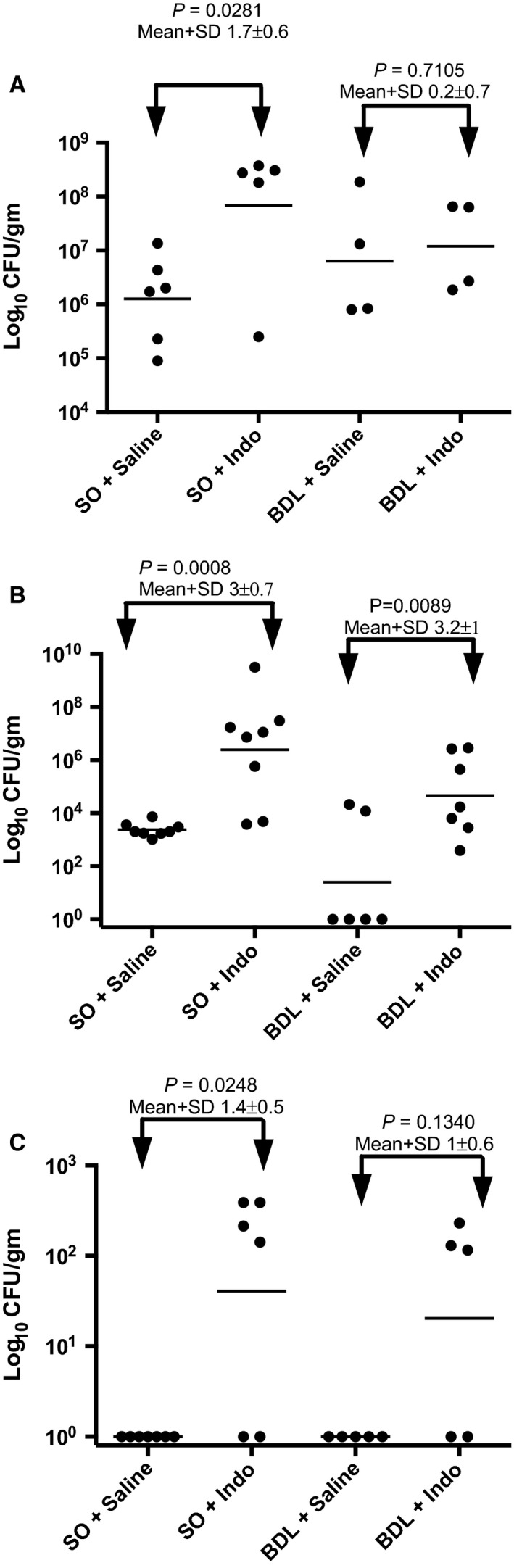
Enterococci were cultured in BEA medium from samples collected at the time of euthanasia. Counts were obtained from: (A) feces, (B) ileum, and (C) liver of animals after parenteral vehicle or indomethacin treatment. The numbers of log_10_ CFU/g per animal are shown as individual dots with the geometric mean for each group shown as a bar. The differences between groups are indicated by bars with arrows.

We studied the effects of indomethacin on the growth of bacteria in the small intestine by utilizing selective and nonselective media for measuring bacterial levels in the feces and ileum. Results with BHI and MAC agars were qualitatively similar to those with BEA, so that only the more selective BEA (enterococci) results are shown here. Enterococcal colony‐forming units (CFU/g) measured in the feces of SO/INDO rats were 100‐fold greater in comparison to SO/saline rats (Fig. 3A; *P* = 0.028). BDL/saline and BDL/INDO rats did not differ from each other and had approximately a 10‐fold nonsignificant increase in CFUs compared to SO/saline. Counts taken from the ileum (Fig. 3B) were again greatly (500‐ to 1000‐fold) elevated in the SO/INDO group when compared to SO/saline rats (*P* = 0.0008). BDL/INDO rats had similar levels of ileal enterococci as SO/saline rats, and somewhat higher levels than BDL/saline. Interestingly, enterococci colonization appeared to be decreased below SO/saline levels in the majority (4 of 6) of BDL/saline rats. Figure 3A and B show that BDL rats were protected against marked gut bacterial overgrowth in response to treatment with indomethacin.

For SO/INDO and BDL/INDO treated mice, 100% of colonies recovered from EA plates (20 CFU from each of 2 rats per treatment group) hybridized to the intragenic *ace* probe, indicating that they were all *E. faecalis* strains. For saline‐treated rats, 7/40 CFUs (17%) from the 2 SO/saline rats and 30/40 CFUs (75%) from the 2 BDL/saline rats hybridized to the *E. faecalis* intragenic *ace* probe. The colonies which did not hybridize to *ace* also did not hybridize to any of the probes specific for *E. faecium*,* E. hirae,* or *E. durans*, indicating that they are other enterococci species.

### Dissemination of enterococci to liver

Possible dissemination of intestinal enterococci was studied by culture of liver tissue for bacterial CFU/g (Fig. 3C). There were no CFUs detected in liver of SO/saline or BDL/saline rats, indicating no contamination during removal of organs. Indomethacin treatment significantly increased dissemination to the liver in SO/INDO, but not BDL/INDO rats, compared to their saline controls.

### Effect of oral indomethacin on intestinal bleeding and bacterial overgrowth

**Figure 4:**
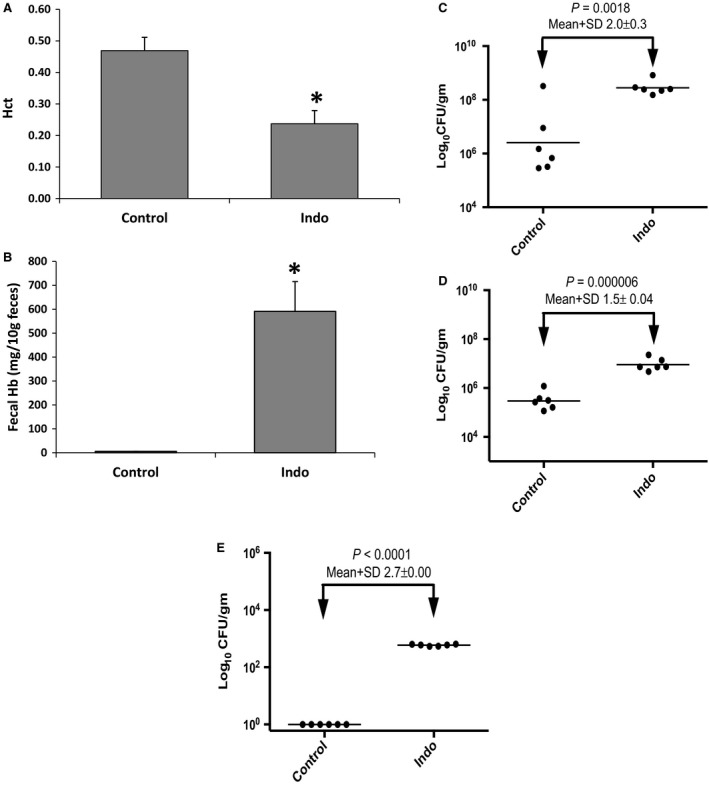
Measurements of: (A) Hct, (B) fecal Hb, and CFU of enterococci cultured from; (C) feces, (D) ileum and (E) liver from animals after oral vehicle or indomethacin treatment. *P<0.05 versus Control.

Indomethacin that was administered orally to rats produced a significant increase in intestinal bleeding seen both as a decrease in Hct (Fig. 4A; *P* = 0.0031) and as an increase in fecal Hb (Fig. 4B; *P* = 0.0014). Measurements of enterococci revealed significant 100‐ to 1000‐fold increases in all tissues (feces, ileum, liver) after oral indomethacin treatment (Fig. 4C–E). All indomethacin animals showed dissemination of bacteria from the gut to the liver.

## Discussion

NSAIDs are often the first‐line treatment of pain and inflammation in many disease processes. Wide availability and chronic consumption contribute to the prevalence of gastrointestinal ulcers and bleeding commonly seen in the clinical setting. Nonaspirin NSAIDs can directly cause damage by changing the hydrophobic nature of the intestinal mucosa (Goddard et al. 1987) and indirectly as well by the increased permeability and bleeding of the gut (Hond et al. 1999), leaving it more susceptible to bacterial growth and dissemination. One possible method to reduce the mucosal damage associated with usage of NSAIDs was thought to be the use of proton pump inhibitors (PPIs) which have been used successfully at reducing NSAID‐induced gastropathy. However, intestinal bleeding is not responsive to PPIs which have a variety of negative side effects when given chronically. Indeed, chronic PPI use in a rodent model resulted in an exacerbation of indomethacin‐induced injury and induced a dysbiosis of intestinal bacteria (Wallace et al. 2011). Other methods to prevent NSAID injury to the intestine that remain to be fully tested include the use of lower‐toxicity COX‐2 inhibitors, prostaglandins, antimicrobials and probiotics (Lim and Chun 2013), and NSAIDs modified by the addition of H_2_S (Wallace et al. 2010, 2015) or phosphatidylcholine (PC) (Lanza et al. 2008; Lichtenberger et al. 2009a,b; Wallace et al. 2010; Blackler et al. 2012; Dial et al. 2015).

A role for bile secretion in the promotion of NSAID‐induced GI injury was demonstrated when it was shown that bile duct ligation could prevent indomethacin‐induced gut injury (Brodie et al. 1970; Somasundaram et al. 1997; Jacob et al. 2007). In addition to the normal presence of bile acids and phosphatidylcholine, bile secretion also contains significant concentrations of INDO due to the enterohepatic cycling of the drug, much of which is processed by the liver into an acyl glucuronide (Boelsterli and Ramirez‐Alcantara 2011) or other metabolites (Yesair et al. 1970; Beck et al. 1990) and then is secreted with the bile fluid (Beck et al. 1990). This enterohepatic circulation redelivers INDO to the small intestine where it is theorized that bacteria containing *β*‐glucuronidase can cleave off the glucuronic acid moiety, increasing exposure of the gut mucosa to unconjugated INDO. Estimates of the amount of INDO secreted through the bile fluid range from 20% to 65% of the initial dose over a 24‐h period (Yesair et al. 1970; Beck et al. 1990). BDL could be blocking a substantial amount of INDO from reaching the intestinal mucosa through the bile, but there is still INDO in the systemic circulation. Additionally, we have proposed that bile acids and INDO can form toxic mixed micelles within the bile fluid that contribute to INDO‐induced GI injury (Zhou et al. 2010; Dial et al. 2015). This possibility is partially supported by others (Jacob et al. 2007) who confirmed that INDO does not induce macroscopic intestinal injury in bile duct–ligated rats, while they also found that an oral dose of a bile acid, when mixed with INDO, failed to produce intestinal permeability changes in BDL rats, suggesting that there is another component of bile secretion that is important for GI injury. It was also reported by Lugea et al. (Lugea et al. 1997) that bile collected from INDO‐treated rats, and not control rats, could induce a decrease in mucosal surface hydrobicity, indicative of an injurious factor in bile fluid. BDL thus prevents bile acid, some INDO and any toxic mixed micelles from reaching the gut lumen and thereby decreases intestinal injury. Because of the complexity of this biliary fluid, it is not possible to ascribe a hierarchy of importance to any of these constituents when all are reduced by BDL. However, it appears that a systemically mediated effect of INDO in the presence of BDL is insufficient to affect small bowel injury and SBO, which supports the theory that it is the mucosal exposure or topical effect that is more important for injury.

Our studies with orally administered indomethacin also provide evidence that a direct exposure of the gut to the drug results in significant GI injury. Gut bleeding was more severe with oral dosing than with parenteral administration, possibly due to a higher drug concentration in the intestine.

A role for intestinal bacteria in the promotion of NSAID‐induced GI injury has been shown previously. Several groups reported that treatment with antibiotics can prevent NSAID‐induced injury (Kent et al. 1969; Satoh et al. 1983; Evans and Whittle 2001). Also, germ‐free rats treated with indomethacin were protected from gut injury (Robert and Asano 1977). However, when bacteria (*E. coli*) were reintroduced to these rats, they became susceptible to this intestinal damage, although the injury was not as severe as in conventional rats. Interestingly, this early paper (Robert and Asano 1977) noted that germfree rats are deficient in secondary bile acids, and that the presence of these more toxic hydrophobic bile acids may be of importance in the induction of NSAID‐induced lower gut injury. More recently, Dalby et al. (Dalby et al. 2006) showed that treatment of rats with INDO resulted in expansion of normal intestinal microorganisms, especially *E. faecalis,* and this change in microbial flora was associated with NSAID‐induced lower gut injury. We partially confirmed this finding with INDO when we recently reported that a single dose of indomethcin (20 mg/kg, sc) was capable of increasing total intestinal bacteria counts (BHI plates) after 24 h (Lichtenberger et al. 2015). We have now fully confirmed the Dalby et al. finding with our hybridization assays where we show that the INDO‐induced increased enterococci are indeed *E. faecalis*. Furthermore, this INDO‐induced overgrowth of *E. faecalis* in the distal gut was independent of the route of administration, as it was observed in animals subcutaneously or orally dosed with the NSAID.

It is a connection between bile acid and intestinal bacteria that was investigated in our present study. It was clear that SO/INDO rats had the highest levels of enterococci and high bile acid in the intestine, whereas all of the BDL animals had greatly reduced intestinal bile acid and lesser enterococci. In fact, in control animals it appeared that BDL reduced ileal enterococci to near zero in four out of six animals. This reduction was not seen in BDL/INDO animals and underscores that enterococci are tolerant of bile acid, but not dependent on it. It should be noted that BDL is also used in a model of cholestasis (Deitch et al. 1990; Slocum et al. 1992) where animals are maintained for 7 days after BDL and exhibit enhanced intestinal bacterial overgrowth and translocation. Our model with BDL was limited to 3 days so that symptoms of cholestasis would be minimal. There was clearly no bacterial overgrowth in the BDL control animals after 3 days.

On the basis of our results, we have demonstrated that restricting bile flow into the intestine helps to attenuate both INDO‐induced damage to the intestinal mucosa as well as bacterial overgrowth and dissemination, an effect of BDL that has not been reported previously. Bile duct ligation did not entirely prevent gut injury as shown by the histologic evidence of some surface injury of ileal villi, nor the presence of enterococci CFUs in liver of some BDL/INDO rats. We cannot rule out the possibility that some of the injury of rats in the BDL/INDO group may be the result of surgical trauma, related to inflammation from manipulation of the gut. Perhaps, what is more important is that BDL helped prevent clinical injury as demonstrated by the minimal hemoglobin detection in feces and preservation of hematocrit levels in BDL rats treated with INDO. BDL/INDO treatment was also consistently associated with reductions of enterococci in the gut tissue, feces, and liver compared to SO/INDO.

In summary, we have demonstrated that subcutaneous injection of indomethacin induces both intestinal injury and enterococci overgrowth/dissemination, and that these changes tend to be reduced with prior bile duct ligation. The isolation and measurement of intestinal bacteria and identification of enterococci as specifically promoted by INDO, as well as the reduction of bacterial growth by BDL are novel findings. It remains unclear whether it is the NSAID‐induced intestinal injury that is causing the bacterial overgrowth or whether the bacterial overgrowth in response to NSAID‐treatment contributes to the injury. Future studies should focus on determining the causal relationship of these factors. The current studies are consistent with previous studies in supporting an important role for both bile acid and bacteria in the pathogenesis of lower gut injury to indomethacin and other nonaspirin NSAIDs.

## Conflict of Interest

Dr. Murray reports grant support from Cubist and Forest, grant support and personal fees from Theravance, and personal fees from Astra‐Zeneca, Rib‐X and GlaxoSmithKline, all outside the submitted work. Dr. Dial reports stock options from PLx Pharma LLC, outside the submitted work. Dr. Lichtenberger reports grant support and stock from PLx Pharma LLC, outside the submitted work. The remaining co‐authors have no disclosures to report.
